# A Case Report and Literature Review of Rectosigmoid Crohn’s Disease: A Diagnostic Pitfall Ultimately Leading to Spontaneous Colonic Perforation

**DOI:** 10.7759/cureus.36941

**Published:** 2023-03-31

**Authors:** Muhammad Z Ali, Muhammad Usman Tariq, Muhammad Hasan Abid, Hamma Abdulaziz, Mohmmad AlAdwani, Arif Khurshid, Muhammad Rashid, Fawaz Al Thobaiti, Amjad Althagafi

**Affiliations:** 1 General Surgery, Alhada Armed Forces Hospital, Taif, SAU; 2 Histopathology, Prince Faisal Cancer Centre, King Fahd Specialist Hospital, Buraydah, SAU; 3 Continuous Quality Improvement and Patient Safety, Alhada Armed Forces Hospital, Taif, SAU; 4 Quality Improvement and Patient Safety Leadership, Institute for Healthcare Improvement, Boston, USA; 5 Colorectal Surgery, Alhada Armed Forces Hospital, Taif, SAU; 6 Surgery, Alhada Armed Forces Hospital, Taif, SAU

**Keywords:** spontaneous colonic perforation, diagnostic pitfall, inflammatory bowel disease, ulcerative colitis, crohn’s disease

## Abstract

Inflammatory bowel disease (IBD) is a chronic condition that affects the gastrointestinal tract, with ulcerative colitis (UC) and Crohn's disease (CD) as the two major entities. While these conditions share some similarities in clinical presentation, they have distinct histopathological features. UC is a mucosal disease affecting the left colon and rectum, while CD can affect any part of the gastrointestinal tract and all layers of the bowel wall. Accurate diagnosis of UC and CD is important for effective management and prevention of complications. However, distinguishing between the two conditions based on limited biopsy specimens or atypical clinical presentations can be challenging. We present a case of a patient diagnosed with UC based on a single endoscopic biopsy from the sigmoid colon, who later presented with colonic perforation and was found to have CD on the colectomy specimen. This case emphasizes the importance of clinical guidelines when dealing with any patient of suspected IBD, considering alternative diagnoses in patients with atypical presentations and the need for careful clinical, endoscopic, and histological evaluation to make an accurate diagnosis. Delayed or missed diagnosis of CD can lead to significant morbidity and mortality.

## Introduction

Inflammatory bowel disease (IBD) is a chronic disorder characterized by inflammation of the gastrointestinal tract, which affects approximately 1.6 million Americans [1]. The two major entities of IBD are ulcerative colitis (UC) and Crohn’s disease (CD), which have distinct clinical presentations and histopathological features [2]. UC is a mucosal disease that starts usually in the rectum and later can involve the whole colon, while CD affects any part of the gastrointestinal tract mouth to anus and can affect all layers of the bowel wall [2]. The diagnosis of UC and CD is typically based on a combination of clinical, endoscopic, and histological findings. However, distinguishing between these two conditions can be challenging, particularly in cases with atypical presentations or limited biopsy specimens.

Histopathological evaluation of biopsy and resection specimens is essential for the diagnosis of IBD, but it should be interpreted in conjunction with other clinical, laboratory, radiological, and endoscopic features. Currently, there is no gold standard modality available for the diagnosis of IBD, making it challenging to differentiate between these two entities [2]. Therefore, a comprehensive approach that incorporates multiple diagnostic modalities is necessary to achieve an accurate diagnosis. Failure to do so may result in delayed or missed diagnoses, which can have significant consequences for patient outcomes [3]. A review highlighted the importance of accurate diagnosis of IBD for appropriate treatment and patient safety [4]. There is evidence that about 3-10% of patients with colonic inflammation have overlapping features, making it difficult to differentiate between UC and CD, and as per one cohort study, in about 10% of patients, a change in the diagnosis was reported after reevaluation during follow-up of patients originally diagnosed with UC or CD [5].

We present a case of rectosigmoid CD, misdiagnosed as UC on endoscopic biopsy, which resulted in treatment failure and presented as spontaneous colonic perforation and generalized peritonitis.

## Case presentation

A 27-year-old female presented to the gastroenterology clinic in June 2018 with a complaint of bloody diarrhea, off-and-on passage of mucus in stools, occasional tenesmus, and marked weight loss of about 25 kg over two months. Her initial laboratory findings revealed low hemoglobin, elevated erythrocyte sedimentation rate (ESR), elevated C-reactive protein (CRP) levels, and positive stool occult blood test. Stool cultures for *Salmonella*, *Shigella*, *Campylobacter*, and *Yersinia* were negative (Table 1).

**Table 1:** Summary of laboratory investigations done at the initial visit to gastroenterology clinic

Laboratory parameters	Result	Normal Range
Hemoglobin	9.6	12-15 g/dL
Erythrocyte sedimentation rate (ESR)	90	0-29 mm/hr
C-reactive protein (CRP)	110	0-5.0 mg/L
Stool for occult blood	Positive	Negative
Stool culture	Negative	-
Varicella-zoster IgG antibody (titer)	Negative (<10.00)	150-4000 mIU/mL
Rubella IgG antibody (titer)	Negative (28)	0-9.99 IU/mL
Brucellosis antibody	Negative	Negative
Sickle cell test (Hb-S screening)	Negative	Negative

**Figure 1:**
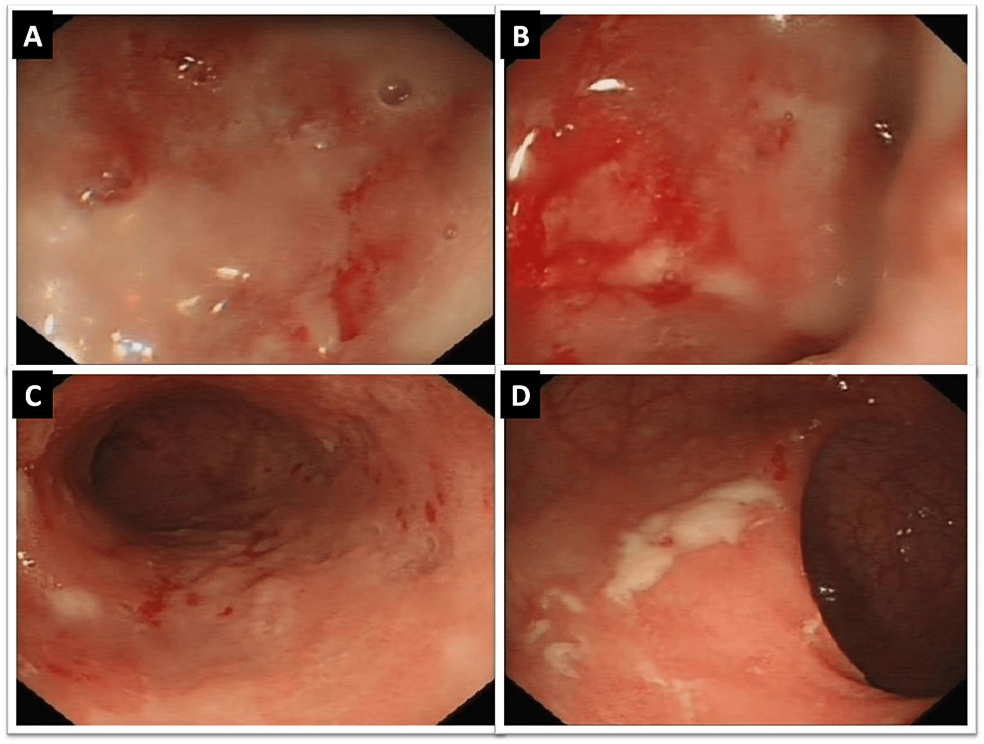
Initial colonoscopy images showing extensive ulcerations and friable vascular mucosa from the anus to the rectosigmoid region

The patient underwent an initial incomplete colonoscopy that was limited to 35cm from the anal verge with findings of extensive ulcerations and friable vascular mucosa from the anus to the rectosigmoid region (Figure 1 A-D).

**Figure 2:**
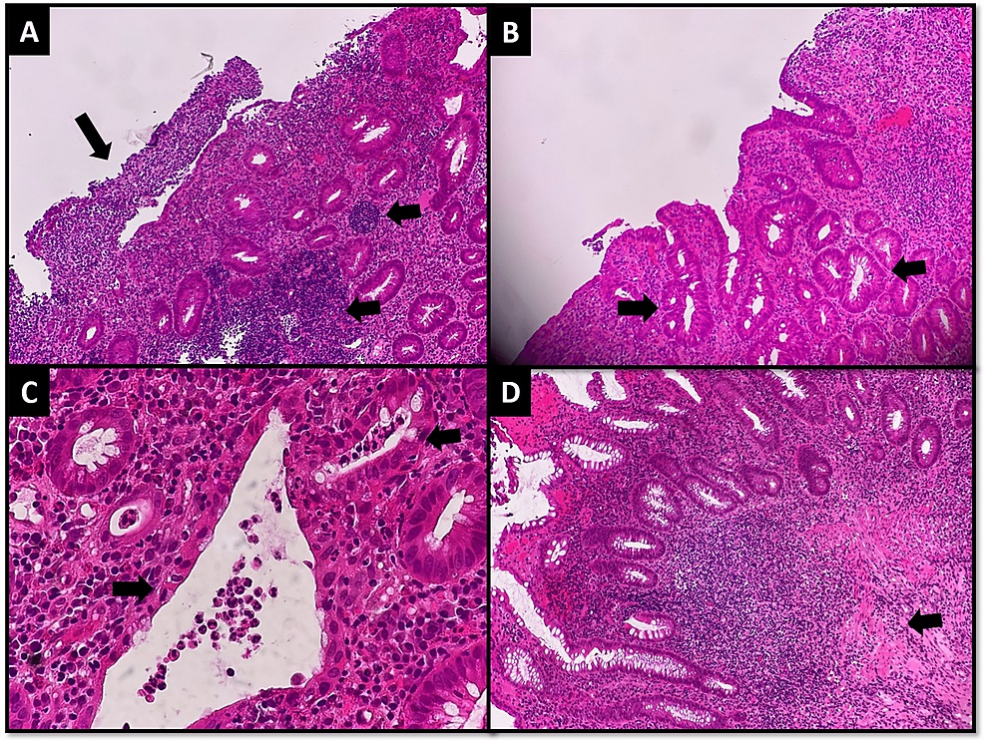
Initial rectosigmoid endoscopic biopsy (A) Large bowel mucosa showing ulceration (larger arrow) and moderate chronic inflammation with lymphoid aggregates (smaller arrows), (B) distortion of crypt architecture (arrows), (C) cryptitis and crypt abscess (arrows), and (D) inflammation extending deeper through muscularis propria (arrows).

No CT scan or MRI scan of the abdomen and pelvis was performed. Microscopic examination of the biopsy showed ulceration, diffuse severe chronic active inflammation, lymphoid aggregates, distortion of crypt architecture, and crypt abscess; but granuloma formation was not observed (Figure 2 A-D).

Based on histological findings, she was diagnosed with UC and started on mesalamine 500mg twice daily and prednisolone 35mg daily. Two months later, azathioprine 100mg daily was added due to persistent symptoms of rectal bleeding and diarrhea. 

The patient initially showed some improvement in her symptoms and she gained some weight as well. Prednisolone was stopped but resumed with azathioprine in February 2019 due to incomplete resolution of rectal bleeding. She continued the treatment and remained under the gastroenterologist’s follow-up every two months. In June 2019, she presented to the hematology clinic due to generalized weakness and fatigue, with off-and-on bleeding per rectum and stool frequency of four to five times a day. Laboratory findings revealed iron deficiency anemia (Table 2).

**Table 2:** Summary of laboratory investigations done at the visit to hematology clinic TSH: thyroid-stimulating hormone

Laboratory parameters	Result	Normal Range
Hemoglobin	9.5	12-15 g/dL
Serum ferritin	11.4	4.6-204 ng/mL
Serum iron	9.7	4.5-27.9 µmol/L
Total iron binding capacity	42	45-81 µmol/L
Serum prolactin	7.5	5.18-26.63 ng/mL
Serum vitamin D	23	30-100 ng/mL
Serum T4	12.04	9.01-19.05 pmol/L
Serum TSH	1.28	0.35-4.94 mIU/L
Serum uric acid	0.23	0.15-0.35 mmol/L
Serum fibrinogen	797	200-400 mg/dL
Fibrin degradation products	0.68	0.00-0.50 µg/mL

She received intravenous iron treatment for three months and followed up in the hematology clinic until March 2020. Her hemoglobin improved to 14.7g/dL and serum ferritin level increased to 101 ng/mL. She also had some improvement in bleeding per rectum episodes and diarrhea. She was advised to visit the gastroenterology clinic for follow-up assessment of UC but did not do so. 

In April 2020, the patient became pregnant. In June 2020, she presented to the emergency department (ED) with rashes and nodules on her elbows and knees. Her gastroenterologist reduced the dose of mesalamine and azathioprine, stopped prednisolone, and added adalimumab 40 mg to the treatment due to her pregnancy. She continued receiving adalimumab 40 mg subcutaneously every other week and delivered a healthy boy in January 2021. Repeat colonoscopy and imaging (CT scan and/or MRI scan) were not performed because the patient was reluctant due to her pregnancy.

In February 2022, the patient again presented to the ED with complaints of generalized abdominal pain for three days, fever, abdominal distension, and constipation for one day. On examination she was in a state of septic shock, her pulse rate was 125 beats per minute, blood pressure was 90/45 mmHg, and temperature was 99 °F. Abdominal examination revealed distension, tenderness, and rigidity with absent bowel sounds. Digital rectal examination did not reveal any growth or ulcer, and external perianal examination did not reveal any fistula opening, fissure, or skin tags. Laboratory findings on admission were elevated CRP, ESR, WBC, and platelets (Table 3).

**Table 3:** Summary of laboratory investigations done at presentation to emergency department

Laboratory parameters	Result	Normal Range
Hemoglobin	9.3	12-15 g/dL
Erythrocyte sedimentation rate (ESR)	43	0-29 mm/hr
C-reactive protein (CRP)	341	0-5.0 mg/L
Blood urea nitrogen	2.6	2.5-6.7mmol/L
Serum creatinine	54	53-97 mol/L
Serum amylase	21	25-125 U/L
Serum lipase	10	8-78 U/L
WBC	38 x 10^9^/L	4-11 x 10^9^/L
Platelets	713 x 10^9^/L	150-410 x 10^9^/L

**Figure 3:**
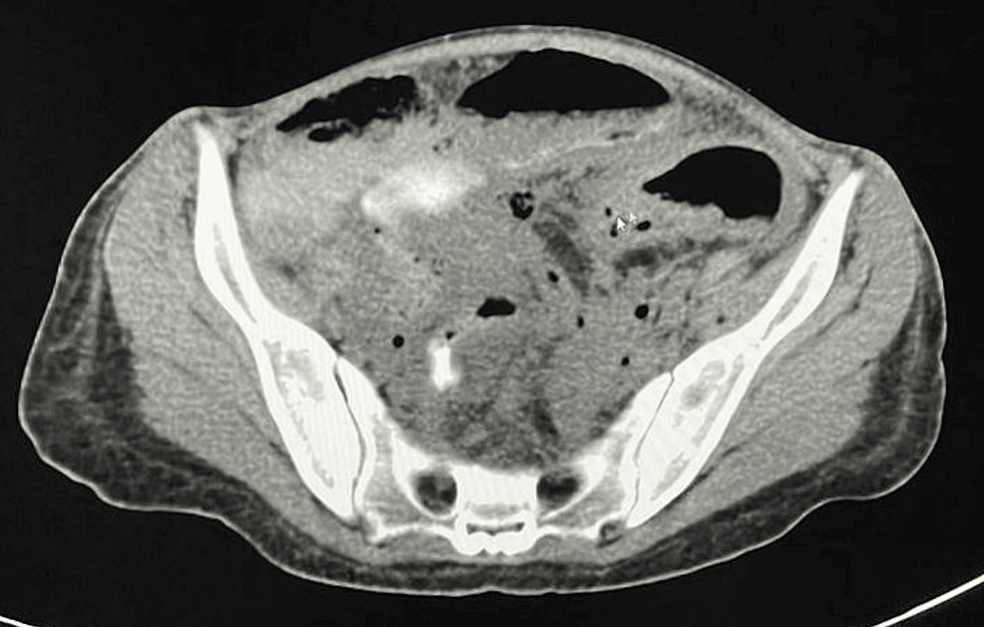
Pre-operative CT scan abdomen and pelvis CT scan with contrast showing pneumoperitoneum, free fluid in the abdomen with septations in the pelvis, and multiple wall-enhancing pockets of collections. Pneumatosis intestinalis is seen in the wall of transverse colon.

The patient was resuscitated with intravenous (IV) fluids and IV ceftriaxone 2g. Her blood pressure improved with fluid resuscitation. X-ray abdomen in erect position showed free air under the diaphragm. A computed tomography scan of the abdomen and pelvis with contrast showed moderate pneumoperitoneum, moderate amount of free fluid in the abdomen with septations in the pelvis, and multiple wall-enhancing pockets of collections. The walls of the transverse colon and ascending colon showed pneumatosis intestinalis. Free flow of contrast was seen through the stomach and small bowel, and minimal passage of contrast was seen in the caecum (Figure 3). A diagnosis of generalized peritonitis most likely secondary to colonic perforation was made and exploratory laparotomy was planned. 

The patient and her family were briefed and counseled about the situation, and informed consent was obtained for total colectomy. Upon entering the abdomen during exploratory laparotomy, around 3-liter foul-smelling dirty fluid and pus were found in the peritoneal cavity. The rectosigmoid area was deformed and a large perforation was identified. The terminal ileum was densely adhered to the rectosigmoid area, forming a mass. The tinea coli of the caecum were split with impending perforation due to the possibility of closed-loop obstruction. 

Keeping in view the diagnosis of UC, total colectomy was performed along with resection of the diseased sigmoid and upper rectum. The rectum was oversewn as a defunctionalized Hartman’s pouch and an end ileostomy was performed. The peritoneal cavity was thoroughly irrigated with 25 liters of normal saline. A pelvic drain was employed, and another soft drain was placed in the rectum to avoid possible rectal stump blowout. 

Her postoperative course remained uneventful. Tachycardia and fever subsided the next day, and ileostomy started to function nicely on the second postoperative day. She also started oral diet from the second postoperative day, and ileostomy output was replaced with normal saline and potassium. Both rectal and pelvic drains were removed on the third postoperative day. WBC on the first postoperative day started to go down to 28 x 10-9/L, and on the fourth postoperative day, the WBC was 15 x 10-9/L. CRP dropped to 199.6 mg/L on the first postoperative day and on the fourth postoperative day, the CRP value dropped down to 139mg/L. The patient was discharged home on the fifth postoperative day, with hemoglobin of 11.2 g/L; all other clinical and laboratory parameters were stable upon discharge. The patient was also advised for an outpatient department (OPD) follow-up after one week to review histopathology results. 

One week post hospital discharge, the patient was seen in the OPD clinic with no active complaints. She was tolerating oral diet and receiving the stoma output replacement with oral fluids, and oral rehydration fluids. Stoma was functioning well and no evidence of any early stoma-related complications was observed. The laparotomy wound was intact and a minimal amount of pus discharge was seen through the lower end of the wound that was cleaned; the patient was instructed to have daily dressings change as an outpatient.

**Figure 4:**
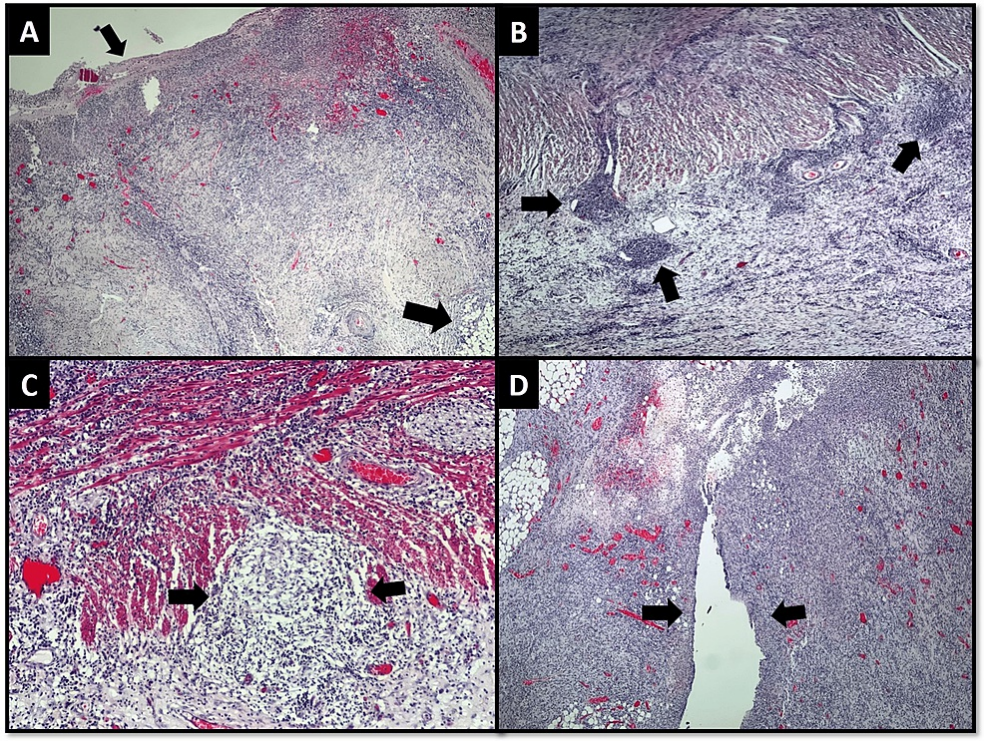
Histological findings of the resection (colectomy) specimen (A) Bowel wall showing ulceration (smaller arrow) and transmural inflammation extending into pericolic adipose tissue (larger arrow), (B) Lymphoid aggregate in the perimuscular and subserosal connective tissue (arrows), (C) Granuloma formation (arrows), (D) Fistulous tract (arrows)

Microscopic examination of the colectomy specimen revealed extensive deep ulceration of the left side of the colon and severe chronic active transmural inflammation. Non-necrotizing granulomatous inflammation, lymphoid aggregates, microabscesses, fistulous tracts, and serositis were also seen (Figure 4 A-D).

Based on the histological findings, the final diagnosis of Crohn’s disease was made. At the 12-month follow-up after the surgery, the patient’s general condition improved significantly as she gained around 15 kg weight and normal functioning stoma. She also underwent terminal ileum and rectal biopsies to assess any active disease. These biopsies did not reveal any significant inflammation.

## Discussion

Both UC and CD can involve the colon, although approximately 10-20% of the time it is impossible to differentiate between these two entities based on clinical or histological grounds, and a diagnosis of indeterminate colitis is made. Similarly, according to some other reports, ambiguous diagnosis is present in 30% of cases of IBD with colonic involvement [6,7]. Correct diagnosis of either condition, UC or CD, is important because surgical treatment and long-term prognosis are different for both [6]. There are clinical practice tools of recommendation and internationally established guidelines for the diagnosis of IBD [2,8].

Little is known regarding the natural history, pathological spectrum, and outcome, particularly after surgery, in patients with isolated CD of the colon, and 14-32% of all cases of CD, at disease onset have isolated colonic involvement [9].

In some cases, the diagnosis of IBD and the distinction between CD and UC is difficult through a mucosal biopsy. The factors which raise this challenge include histological mimics of IBD, insufficient clinical details, unreliable microscopic features, absence of histological changes in early IBD, and atypical clinical presentation of IBC forms [10]. One of the criteria to distinguish UC from CD is superficial versus transmural inflammation but because of the superficial nature of endoscopic biopsy, it is not routine practice to definitely diagnose UC from CD in the initial colonic biopsy. The depth of ulceration (mucosal vs deeper than mucosa) may be helpful in some biopsy specimens to suggest the diagnosis of UC over CD [11]. In most situations, the presence of diffuse and chronic active colitis, with universal involvement of the rectum and absence of granulomas are the key histological features that favor the diagnosis of UC [11]. In a study conducted on endoscopic findings in CD patients, granuloma formation was observed only in 10/41 (24.4%) cases [12]. Patients with isolated colonic involvement demonstrate a lower number of major microscopic features of CD. These patients present with higher age at diagnosis, shorter duration of colitis symptoms, and higher proportion of subtotal, total, and left-sided colitis. These patients exhibit a significantly lesser frequency of strictures, pericolonic adhesions, pyloric metaplasia, and more severe proximal disease than the distal disease. A small proportion (13-14%) of these cases have superficial (mucosal) inflammation without transmural inflammation, which is termed “ulcerative colitis-like CD” [9]. Because of these limitations in distinguishing between the IBD forms, it is mandatory to correlate with clinical, histopathological, radiological, endoscopic, and serological findings [11]. Since the clinical, colonoscopic, and histopathological findings were going in favor of UC and there was no indication of atypical CD at initial presentation, the need for performing imaging studies was not felt by the gastroenterologist, and the treatment for UC was initiated.

Endoscopic examination of the colon is mandatory in three to six months, whenever any treatment is initiated for UC or if treatment is switched to another type, particularly when treatment with a biological agent is decided for the patient [6]. Our patient was diagnosed as UC by the gastroenterologist based on symptoms of rectal bleeding, weight loss, diarrhea, and exclusion of infectious causes based on laboratory findings and endoscopic biopsy results. However, the patient did not have a follow-up colonoscopy during her treatment despite changing treatment regimens. MRI scan of the small bowel is usually reserved for small bowel CD, and only in selected cases to follow up the disease course. CT abdomen pelvis is usually performed when the patient is admitted with features of toxic megacolon either because of UC OR CC [13]. She also lost follow-up of the gastroenterology team for almost one year in 2021. Moreover, when she presented to ER for the first time in 2021, the gastroenterologist considered repeating the colonoscopy and performing imaging but the patient refused because of her pregnancy.

She presented for the first time under general surgery care in ER, with fulminant peritonitis and sepsis, already under treatment for a diagnosis of UC and on biological therapy. Keeping in view the background diagnosis of UC (favored on previous histopathology), with colonic perforation, and the possibility of development of carcinomatous change (although too early), we decided on total colectomy and resection of the ileal part that was adhered to the mass. Final postoperative histopathological findings were consistent with CD. In our patient's case, it is unclear whether UC progressed to CD or was misdiagnosed on the initial rectosigmoid small biopsy. The histological distinction between UC and CD is usually not possible on a small biopsy specimen unless granuloma formation is seen which favors CD. In the context of the initial clinical, radiological, colonoscopic, and histopathology reports, the gastroenterologist made a diagnosis of UC but failed to repeat the colonoscopy and biopsy despite repeated visits to the gastroenterology clinic.

Upon review of the literature, we found some reports in which UC transformed to CD [14,15]. In 1970, Kent et al. introduced the term intermediate colitis due to overlapping features of UC and CD [16]. As per a case report by Yadav and Zhang, UC can progress to intermediate colitis or from intermediate colitis to CD since the features of intermediate colitis are intermediate to both UC and CD [15]. In one prospective study conducted by Andersen et al. on 513 cases, 28.7% of the cases of UC showed progression to CD while 23.9% of cases of CD showed changes in disease localization. The authors of this study hypothesized that UC may progress to CD, while CD cannot [17]. In another study from Korea on 1444 patients of UC, the final diagnosis turned out to be intermediate colitis in 0.4% and CD in 1.7%. [5]. Although all these studies raise the possibility that CD is a continuum of UC and not a different entity of IBD, and UC can be transformed into CD, we believe that this is not the situation in our case. In our patient, the first colonoscopy was only limited to the rectosigmoid area, no full colonoscopy with ileal intubation was done. Secondly, she never had a repeat colonoscopy despite her changing clinical situations and medication regimens. So, we don’t have real past data to say that it was a transformation to CD, or that she was misdiagnosed from the start. Perianal CD occurs in almost 40% of cases of Crohn’s colitis. Interestingly, our patient never developed perianal features of Crohn’s colitis like anal fistulas, fissure in ano, etc., even though she had isolated rectosigmoid disease. 

Free peritoneal perforation, one of the rare conditions of IBD, is a serious event that occurs in 1-3% patients of CD patients either during the course of the disease or as the first manifestation [18,19]. A possible proposed mechanism of free colonic perforation in CD is upstream colonic dilatation. Another mechanism is Crohn’s colitis causing the development of inflammatory changes in the blood vessels that subsequently lead to ischemia and then perforation [20]. 

The management of colonic perforation in CD varies and depends upon whether perforation is due to toxic megacolon or segmental colitis. In the case of toxic megacolon and perforation, total colectomy and end ileostomy is the treatment of choice and segmental resection is the preferred treatment in case of isolated segmental Crohn’s colitis with perforation [21]. 

In our patient, we did total colectomy because of two reasons: first of all, when she presented to general surgery care for the first time, she was a known case of UC for three years and was already under treatment, so total colectomy is the treatment of choice in such situation. Secondly, because of the stricture in the rectosigmoid area, she had gross upstream colonic dilatation with impending cecal perforation.

## Conclusions

This case highlights the importance of following clinical guidelines when dealing with any patient of IBD, considering alternative diagnoses in patients with atypical clinical presentations or limited biopsy specimens. Distinguishing between UC and CD can be challenging, and careful consideration of the clinical, endoscopic, and histological findings is necessary to make an accurate diagnosis. Delay in the diagnosis or misdiagnosis can lead to significant morbidity and mortality, and appropriate management requires a prompt and accurate diagnosis.
